# Systematic cross-validation of 454 sequencing and pyrosequencing for the exact quantification of DNA methylation patterns with single CpG resolution

**DOI:** 10.1186/1472-6750-11-6

**Published:** 2011-01-14

**Authors:** Anna Potapova, Cord Albat, Britta Hasemeier, Katrin Haeussler, Stella Lamprecht, Sebastian Suerbaum, Hans Kreipe, Ulrich Lehmann

**Affiliations:** 1 Institute of Pathology, Medizinische Hochschule Hannover, D-30625 Hannover, Germany; 2 Institute of Biometrics, Medizinische Hochschule Hannover, D-30625 Hannover, Germany; 3 Institute of Medical Microbiology and Hospital Epidemiology, Medizinische Hochschule Hannover, D-30625 Hannover, Germany

## Abstract

**Background:**

New high-throughput sequencing technologies promise a very sensitive and high-resolution analysis of DNA methylation patterns in quantitative terms. However, a detailed and comprehensive comparison with existing validated DNA methylation analysis methods is not yet available. Therefore, a systematic cross-validation of 454 sequencing and conventional pyrosequencing, both of which offer exact quantification of methylation levels with a single CpG dinucleotide resolution, was performed.

**Results:**

To this end the methylation patterns of 12 loci (*GSTπ1, p16*^*INK4a*^*, RASSF1A, SOCS1, MAL, hsa-mir-1-1, hsa-mir-9-3, hsa-mir-34a, hsa-mir-596, hsa-mir-663, MINT31*, and *LINE-1*) were analyzed in ten primary hepatocellular carcinoma specimens. After applying stringent quality control criteria, 35749 sequences entered further analysis. The methylation level of individual CpG dinucleotides obtained by 454 sequencing was systematically compared with the corresponding values obtained by conventional pyrosequencing. Statistical analyses revealed an excellent concordance of methylation levels for all individual CpG dinucleotides under study (r^2 ^= 0.927).

**Conclusions:**

Our results confirm that 454 sequencing of bisulfite treated genomic DNA provides reliable high quality quantitative methylation data and identify *MAL, hsa-mir-9-3, hsa-mir-596, and hsa-mir-663 *as new targets of aberrant DNA methylation in human hepatocelluar carcinoma. In addition, the single molecule resolution of 454 sequencing provides unprecedented information about the details of DNA methylation pattern heterogeneity in clinical samples.

## Background

Traditionally, cancer has been regarded as a disease that is driven by progressive genetic abnormalities including mutations and chromosomal aberrations in tumor-suppressor genes and oncogenes. However, it has become clear that cancer is also driven by 'epigenetic changes' -- patterns of altered gene expression that are mediated by mechanisms that do not affect the primary DNA sequence [1]. The main epigenetic mechanisms are DNA methylation, histone modifications and non-coding RNAs. DNA methylation represses transcription directly, by inhibiting the binding of specific transcription factors, and indirectly, by recruiting methyl-CpG-binding proteins and their associated repressive chromatin remodeling activities [2]. Since the hypermethylation of CpG islands is relatively rare in normal cells, and often an early event in transformation, it represents a promising biomarker for early cancer detection [3].

The methylation status of DNA can be detected by several methods [4]. To measure methylation levels, bisulfite conversion has been combined with restriction analysis (COBRA [5]), base-specific cleavage and mass spectrometry [6], real-time PCR (MethyLight, [7]), and pyrosequencing [8]. However, not all of these methods provide quantitative data with a single CpG resolution. The technique considered by many in the field as the gold standard is bisulfite genomic sequencing, which examines multiple subclones of a bisulfite PCR product [9]. However, if only 5 - 10 clones are sequenced (as in most published studies) this approach is at best semi-quantitative and not very sensitive.

Over the past five years, DNA sequencing technology has evolved rapidly. Among others, a novel massively parallel sequencing-by-synthesis method was introduced that is based on pyrosequencing in picoliter-scale reactions ("454 sequencing"). The 454 technology generates ~ 400,000 reads per instrument-run at lengths of 200 to 300 bp with an accuracy of 99.6% [10]. With the introduction of new reagents the average read length is increased to 400 - 500 bp [11]. In principle, the large number of sequence reads per amplicon (dozens or even hundreds) offers the opportunity to obtain precise quantitative methylation data for every single CpG site contained within the amplicon. To the best of our knowledge, this has not yet been studied comprehensively employing rigorous statistical tools.

Therefore, we cross-validated 454 amplicon bisulfite sequencing and conventional pyrosequencing, which is a well established and validated, highly quantitative method for the exact quantification of DNA methylation patterns with single CpG resolution by measuring the methylation level of 89 CpG dinucleotides from 12 loci in 10 patient samples.

## Results

### Selection of loci

In order to explore the feasibility of quantitative methylation studies using 454 sequencing, a range of genomic loci was selected for in-depth analysis: tumor-suppressor genes reported to be frequently hypermethylated in hepatocellular carcinoma (*p16*^*INK4a*^*, RASSF1A, SOCS-1, GSTπ1*, [12] and references therein), classical non-protein coding CIMP loci also reported to be methylated in HCC (*MINT31*, [13]), and microRNA genes identified by our own group to be aberrantly methylated in HCC (*mir-9-3, mir-34a, mir-596, mir-663*). Since Datta et al. [14] reported aberrant hypermethylation of *mir-1-1 *in HCC, this microRNA was also included in the analysis. In addition, LINE-1 sequences were analyzed because these repetitive elements often display marked hypomethylation and a high sequence heterogeneity which might be ascertainable by a deep bisulfite sequencing approach.

### Mapping the bisulfite sequencing results

A total of 59,366 sequences were obtained in a single run using a fraction of a small 454 sequencing plate (25×75 PicoTiterPlate). The maximum expected number of reads for this setting is about 70,000. The average read length was 210 bp (range, 53-325 bp). Bioinformatic analysis consisted of the following three steps: (a) matching each sequence to a unique tagged primer, (b) mapping the amplicon sequences to the *in silico *bisulfite-converted genomic sequence, and (c) compiling sequence identity, sodium bisulfite conversion efficiency and the methylation state for each CpG site. (see Materials and Methods). Of the 59,366 sequences, 50,118 (84.4%) were mapped to a unique amplicon. The two main sequence errors observed were the detection of unconverted (or only partially converted) sequences in individual reads and small deletions in homopolymeric stretches.

On average, 298 sequence reads were obtained for each amplicon after filtering. However, the yield per amplicon was quite variable. The number of reads per amplicon ranged from 0 to 1487 (mean: 297.9, median: 238.8). A detailed analysis of the number of reads obtained (after filtering) for each locus in all 10 samples under study after filtering can be found in Additional File 1. This variation may be caused by the secondary structure of each amplicon, amplicon length, GC content, overall methylation status, and the number of homopolymers present after bisulfite treatment which all effect the linker ligation and emulsion PCR as well as the efficiency of single molecule sequencing reaction. Statistical analysis revealed a significant influence of the amplicon length on the mean number of reads (r = -0.81). In contrast, the tendency for secondary structure and the GC content show only a weak or no influence on the number of reads (see Additional File 2).

### Quantitative DNA methylation analysis of individual CpG islands

**Figure 1:**
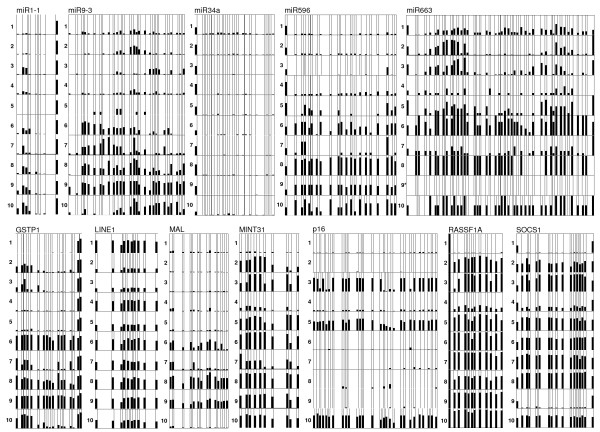
**Methylation profile of 12 loci in 10 HCC samples**. Each *column *represents a CpG site in each DNA sequence. The names of the genes are listed above of each *set*. The sample number is labeled on the left side of each row. The color indicates the methylation level. *White*, no methylation; *black*, methylation. The proportion of *black *and *white *in each *column *indicates the percentage of sequences containing the methylated CpG sites at this position. The sequencing result of one sample (marked by an asterisk) did not pass the quality control (see Materials and Methods) and was not included in the figure.

Based on the quality of the alignment, reads with a sequence identity of less than 90% and less than 100% sodium bisulfite conversion were filtered out. After filtering, a total of 35,749 sequences (60.2%) were used for computing the methylation level at each individual CpG dinucleotide. The percentage of methylation at each CpG site was calculated based on the number of sequences containing unconverted cytosine (indicating methylation in the original sequence) versus the total number of sequences analyzed (Figure 1).

Overall our results for *GSTπ1, p16*^*INK4a*^*, RASSF1A, SOCS1 *and *MINT31 *are consistent with what others have previously reported for HCC (Table 1) [12,13,15-18]. The methylation results for *LINE-1 *elements, *hsa-mir-1-1 *and the newly identified targets *MAL, hsa-mir-9-3, hsa-mir-596*, and *hsa-mir-663*, are also compiled in Table 1.

**Table 1:** Methylation profile of all loci across the 10 HCC samples analyzed in this study.

	RASSF1A	SOCS1	MINT31	LINE1	GSTP1	P16	MAL	miR596	miR663	miR9-3	miR1-1	miR34a
1	10%	8%	4%	61%	9%	3%	6%	5%	21%	10%	11%	7%

2	75%	50%	53%	20%	22%	2%	2%	7%	19%	10%	10%	5%

3	81%	79%	67%	20%	21%	52%	2%	4%	24%	8%	18%	3%

4	28%	24%	15%	54%	11%	4%	6%	12%	10%	8%	15%	6%

5	75%	62%	64%	30%	10%	46%	2%	19%	27%	4%	10%	4%

6	88%	61%	76%	54%	74%	1%	30%	50%	79%	25%	20%	2%

7	89%	71%	37%	42%	28%	1%	12%	16%	18%	28%	17%	3%

8	66%	80%	74%	54%	53%	2%	61%	81%	75%	36%	22%	4%

9	84%	8%	64%	53%	67%	1%	38%	50%	N/A	58%	24%	3%

10	91%	59%	59%	38%	35%	59%	2%	55%	54%	31%	30%	2%

The percentages are the mean methylation values for a given locus in an individual sample (454 sequencing result).

In order to identify allele specific methylation SNPs were identified in the regions under study. Only for *GSTπ1*, *miR-663*, *p16*^*INK4a*^, and *RASSF1A *could potentially informative SNPs be found. C/T polymorphisms were excluded because of the bisulfite treatment and small deletions/insertions were excluded because of difficulties discriminating between true deletions/insertions and 454 sequencing errors. The remaining (small) number of SNP sites did not reveal anything about allele specific methylation.

### Systematic comparison of 454 sequencing with conventional pyrosequencing

Conventional pyrosequencing and 454 sequencing can both be used to quantify the methylation level at every CpG dinucleotide contained within a given sequence. Since no systematic cross-validation of these two methods has been performed so far and only limited data are available about the reliability of 454 sequencing for the purpose of exact quantification of methylation levels (see Discussion), a detailed comparison of the quantitative methylation profile of all 12 loci in all 10 samples was performed. To this end, pyrosequencing assays were designed in such a way that the sequence to analyze was contained completely within the amplicon analyzed by 454 sequencing. Bisulfite treatment for 454 sequencing and conventional pyrosequencing was performed independently for all 10 tumor samples under study.

**Figure 2:**
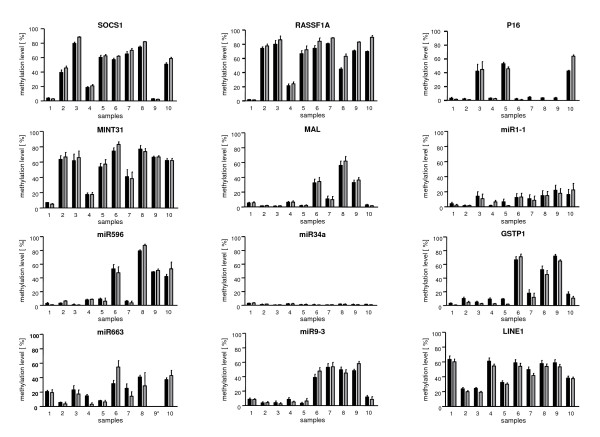
**Cross-validation of 454 sequencing and conventional pyrosequencing: mean methylation level**. Comparison of mean methylation levels for all genes in all samples: pyrosequencing and 454 sequencing data show an excellent congruence. The sequencing result of one sample (marked by an asterisk) did not pass the quality control (see Materials and Methods) and was not included in the figure. Grey columns: 454 sequencing, black columns: pyrosequencing

**Figure 3:**
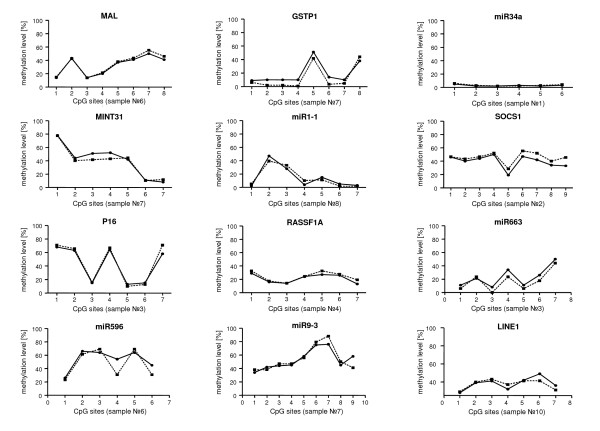
**Cross-validation of 454 sequencing and conventional pyrosequencing: individual methylation levels**. Comparison of the methylation levels of individual CpG dinucleotides of selected genes in selected samples: Even samples with a very heterogeneous methylation pattern (e.g. *p16*^*INK4A*^) display an excellent congruence. (■) 454 sequencing, (●) pyrosequencing

Overall, the mean methylation levels obtained by pyrosequencing and 454 sequencing showed an excellent correlation for every gene in all samples (Figure 2). Also a comparison of the methylation levels of individual CpG sites showed an excellent concordance between both methods for all loci under study (Figure 3).

**Figure 4:**
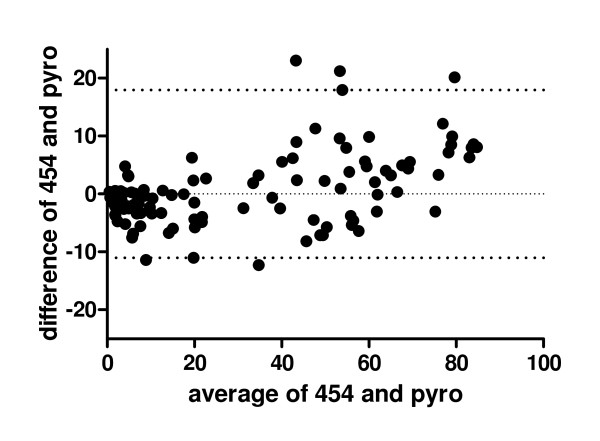
**Comparison of pyrosequencing and 454 sequencing using Bland-Altman Plots**. Bland-Altman-Plot: Difference in methylation level obtained by both methods versus the mean of the methylation level for all 12 loci in all 10 samples. The individual Bland-Altman-Plots can be found in the Additional File 3. The upper and lower dotted lines indicate the 95% tolerance interval.

In Figure 4) a Bland-Altman-Plot is shown to demonstrate the congruence of both methods. In Additional File 3 the Bland-Altman-Plots for each individual locus is shown. A difference of less than 10 percentage points was judged to be acceptable considering the technical variability of conventional pyrosequencing which is in the range of 2 - 10 percentage points [19]. The 95% tolerance intervals for 10 out of 12 loci are well within the range of +/- 10 percentage points.

**Figure 5:**
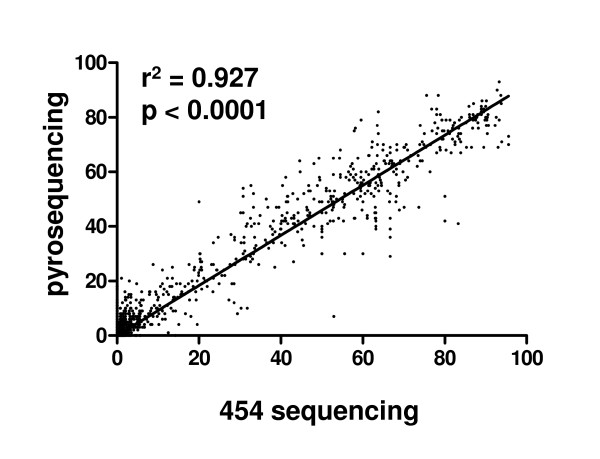
**Comparison of pyrosequencing and 454 sequencing using regression analysis**. Regression analysis was performed for all individual measurements (methylation level of each individual CpG site under study, n = 869) obtained by the two methods. The excellent concordance of both methods is obvious (r^2 ^= 0.927, p < 0.0001, slope: 0.918, 95% confidence interval: 0.9067 to 0.9300).

Regression analysis of the methylation levels of all individual CpG sites under study obtained independently by the two methods revealed a very good concordance (Figure 5, r^2 ^= 0.927, slope: 0.918, 95% confidence interval: 0.9067 to 0.9300). A regression analysis of the mean methylation levels of all samples, which diminishes the influence of outliers, results in an even better concordance (r^2 ^= 0.957, slope: 0.9317, 95% confidence interval: 0.9078 to 0.9556, data not shown). The results of the regression analysis performed for all 12 loci individually are shown in Additional File 4.

Calculating the differences in relative methylation obtained by the two methods for each individual CpG site under study, revealed that for the vast majority of measurements (720/869, 82.8%) a good congruence (i.e., less than 10 percentage points deviation) was observed. 514 out of 869 measurements (59.1%) gave a very good congruence (i.e., less than 5 percentage points deviation), 288/869 measurements (33.1%) a perfect match with less than 2 percentage points deviation. For more details see Additional File 5.

**Figure 6:**
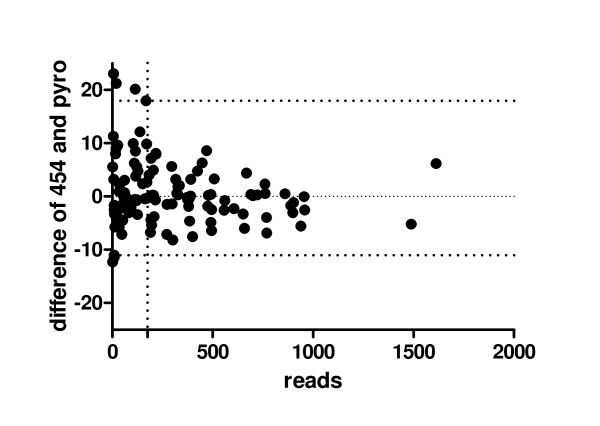
**Relationship between congruence and coverage**. The difference between both methods (as a measure of congruence) was plotted against the number of reads obtained by 454 sequencing. Above coverage of 175 reads per sample no difference lies outside the 95% confidence interval.

Analysis of the *miR-663 *gene gave the worst congruence between pyrosequencing and 454 sequencing. The most likely reason for this is the very low number of 454 sequence reads obtained for this locus. This low efficiency is most probably due to the length of the amplicon (with 312 bp the longest in this study), the tendency to form secondary structures (see Additional File 1) and the extremely high GC content of this locus (%GC: 62.2%, CpG_obs_/CpG_exp_: 0.94), which also made the design of a conventional pyrosequencing assay difficult. One sample (HCC 9) for *miR-663 *was left out from analysis because the quality control criteria of the methylation data analysis software were not met (see also legend to Figure 2). Sample 8 and 10 also yielded only 2 sequences each, and were left out from further statistical analyses. Therefore, the total number of CpG sites under study is 869 (and not 890). Figure 6 shows clearly a correlation between number of reads per amplicon and the congruence of both methods: High read numbers result in better congruence. The samples with a difference between both methods outside the 95% confidence interval all have low read numbers (below 175).

Therefore, a minimum of 100 - 150 reads per amplicon should be achieved for proper quantification of methylation levels be 454 sequencing. Using a full GS20 plate approximately 30 genes in 100 samples could be analyzed in a single run. In contrast to pyrosequencing, which provides an average methylation level for each CpG dinucleotide across a possibly very heterogeneous population of individual DNA molecules, 454 sequencing delivers a single molecule resolution. This reveals, for example, that only very few completely methylated *LINE-1 *alleles can be found, whereas for the *MINT1 *locus a substantial amount of individual alleles is fully methylated (Additional File 6). Other genes display a remarkable heterogeneity in the pattern of DNA methylation across samples: *RASSF1A *for example displays in some samples (with very few exceptions) only fully methylated or fully unmethylated alleles whereas in other samples a much more diverse pattern is found (Additional File 7).

## Discussion

Large scale methylome studies become more and more important in biomedical sciences and high throughput sequencing technologies promise large scale sensitive, quantitative and high resolution DNA methylation analysis. However, the reliability of the quantification of the methylation level of individual CpG sites by these new sequencing technologies has not been explored comprehensively. Therefore, we compared massive parallel bisulfite sequencing using 454 technology with conventional pyrosequencing. The reliability of conventional pyrosequencing has already been demonstrated in several comparative studies by comparing the results with the output of other methodologies (e.g., SnaPShot [20] or COBRA [5] or by analyzing samples with defined methylation status, like cell line DNA [21]. The reproducibility of conventional bisulfite pyrosequencing technology proved to be excellent [8,22].

Despite the fact that several studies already describe the use of 454 bisulfite sequencing for quantitative analysis of methylation data (see below) no comprehensive comparison of these methodologies has been performed so far:

In their pioneering study, Taylor et al. [23] compared methylation data from 454 sequencing, qMSP and conventional bisulfite sequencing for three genes. The data were obtained from the analysis of three sample pools and *in vitro *methylated DNA (Figure 3 in Taylor et al.). Since no statistical analysis is presented in this publication, the extent of congruence seems to be judged by visual inspection of the figures. Ordway et al. [24] compared the mean methylation level obtained by 454 sequencing with the relative methylation density measured by real-time PCR for four genes and found a reasonable correlation (r^2^: 0.45 - 0.87) with respect to this average value (Figure 5, A, C, E, G in Ordway et al.). Xie et al. [25] also compared the mean methylation level obtained by 454 sequencing for various Alu elements with the mean methylation level obtained by conventional pyrosequencing (Figure 4 in Xie et al.). For six loci sufficient data were available for a comparison. The outcome was quite variable, from perfect congruence to clear discordance (e.g., 25% versus 65%). One reason might be the sequence heterogeneity even within subgroups of Alu elements [26]. Varlay et al. [27] compared 454 sequencing data to COBRA results from the same sample for 14 tumors. All samples classified as "methylated" by COBRA had a substantial proportion of molecules (> 15%) with dense methylation in the proximal promoter. The authors defined "dense methylation" as "greater than 50% of CpGs methylated" (Table 1 in Varley et al.). Since no details of the evaluation of the COBRA data are given and no primary COBRA data are displayed, a comprehensive judgment of the degree of congruence is not possible. Also, no comparison for individual CpG dinucleotides has been performed. Korshunowa et al. [28] describe no cross-validation in their study, but this manuscript is from the same group as the above cited publication from Ordway et al. [24]. Therefore, the results are indirectly linked to the comparison described in the latter study. Zeschnigk et al. [29] compared the overall methylation pattern of selected CpG islands obtained by 454 sequencing with the results of conventional bisulfite sequencing. Since they were interested in global methylation patterns, a systematic comparison of methylation levels of individual CpG dinucleotides was not performed. Further, the low coverage achieved in this study (less than 10 reads for the vast majority of CGIs and approx. 10 cloned PCR fragments per locus) did not permit a precise quantification with single-CpG resolution. Hodges et al. [30] compared high throughput sequencing data obtained by an Illumina GA2 platform with conventional bisulfite sequencing of individual clones. For the majority of the CpG sites under study the 90% confidence intervals overlap, but the deviation of the calculated methylation levels is much larger than in this study (supplementary Table two in Hodges et al.). However, these data are not directly comparable since sample preparation, primary output and subsequent data processing are quite different from the 454 sequencing platform.

One limitation of 454 sequencing is the accurate resolution of homopolymer sequences. Because there is no terminating moiety preventing multiple repeated incorporations at a given nucleotide injection cycle, the length of a homopolymeric stretch must be inferred from the signal intensity. This is prone to a greater error rate than the discrimination of incorporation versus non-incorporation [31]. However, the quantification tool for methylation analysis used in this study (QUMA, [32]) was able to accurately align the fragments even if there were gaps present in a homopolymeric region. A few amplicons did not produce a sufficient number of high-quality reads for the quantification of cytosine methylation (i.e., *miR-663*). This may be due to the amplification bias at the library preparation step, resulting in a bias of the fragments generated during the emulsion PCR reaction. However, an insufficient number of reads was obtained for only 3 of the 120 amplicons (2.5%).

A clear advantage of 454 sequencing is the higher sensitivity for the detection of low level methylation. The 1.4% of fully methylated LINE-1 alleles detected in sample no. 8 (see Additional File 6) are not detectable by conventional pyrosequencing due to the inherent background. Depending on the quality of the assay the background signals are in the range of several percentage points. Since the vast majority of LINE-1 sequences are more than 90% similar to each other in the CpG islands contained within the 5'-UTR, a detailed analysis of the different LINE-1 family members would require the amplification and sequencing of much larger regions. However, these other, more diverse regions do not qualify as CpG islands and are most likely not directly affected by differential methylation.

Our data demonstrate that the two different methodologies yielded very similar methylation values obtained for the large majority of CpG sites (82.8%) even for very heterogeneous methylation patterns as exemplified by the *p16*^*INK4a *^gene (range of methylation levels of individual CpG dinucleotides: 10 - 70%, see Figure 3). Further, for low level methylation levels as seen in *miR-34a*, a very high congruence was achieved. This remarkable concordance serves as a powerful validation for both technical approaches which has not previously been demonstrated.

## Conclusion

The 454 technology enables a much more comprehensive coverage of whole CpG islands and the single molecule resolution provides unprecedented information about the heterogeneity of methylation patterns (a few examples are provided in Additional File 7). It also offers the possibility of studying many loci in parallel. These advantages come with a considerable initial investment, expansive reagents, low turn-around time, and a time-consuming data processing and evaluation step, making it primarily a research tool. By contrast, conventional pyrosequencing is generally much faster, easier and cheaper. Provided that only a single locus has to be analyzed at regular intervals, and that the differentially methylated region of interest is well characterized and quite small, as is the case for routine diagnostic applications (e.g., analyzing *MGMT *gene methylation in glioblastoma [33] or *hMLH1 *gene methylation in colorectal carcinoma [34]) conventional pyrosequencing remains the method of choice.

Therefore, in our opinion conventional pyrosequencing and 454 sequencing are not competing but complementary methodologies fulfilling different functions in the field of DNA methylation analysis.

## Methods

### Tissue specimens and bisulfite modification of DNA

All hepatocellular carcinoma (HCC) samples were retrieved from the archive of the Institute of Pathology, Hanover Medical School (Germany) and analyzed anonymously following the guidelines of the local Ethics committee ("Ethik-Kommission der Medizinischen Hochschule Hannover", head: Prof. Dr. Tröger). Tumor cell content was determined to be greater than 70%. DNA was isolated by digestion with proteinase K (Merck, Darmstadt, Germany) followed by phenol/chloroform extraction from a total of 10 specimens (Additional File 8). Genomic DNA (1 μg) from tumor specimens was treated with sodium bisulfite using the Imprint™ DNA Modification Kit (Sigma, Saint Louis, Missouri) following the protocol supplied by the manufacturer.

### Primer design and PCR

Specific primers were designed for 12 loci using publicly available software (http://www.genelink.com). A ten or nine-nucleotide sample-specific tag was added to the 5' end of each forward primer sequence so that each sample could be computationally separated after 454 sequencing analysis. The tag sequences were provided by Roche (Mannheim, Germany). PCR was performed for 33 - 36 cycles in a 50 μl reaction using annealing temperatures from 60°C to 65°C, depending on the locus under study. A complete list of the 120 primer pairs is available in Additional File 9. Denaturation (95°C), annealing, and extension (72°C) times were 30 s, 45 s, and 1 min, respectively. Each amplicon was individually prepared, gel purified, and quantified by Quant-IT PicoGreen kit (Invitrogen, Eugene, Oregon).

### 454 sequencing

For a single 454 sequencing run one hundred twenty amplicons were pooled in equimolar amounts in a single tube. For some amplicons a precipitation step was necessary to increase the concentration. This was performed following standard procedures using ethanol and sodium acetate and glycogen (Sigma, Saint Louis, Missouri) as a carrier. The emulsion PCR and subsequent sequencing reaction were performed exactly as described in the GS FLX emPCR Method Manual (USM-00033.A, Roche, Mannheim, Germany).

One region of a PicoTiterPlate (25×75) was used with GS FLX-chemistry. Using this configuration the expected yield is approx. 70,000 reads and 17.5 Mbp sequence content in total.

### Methylation analysis using Pyrosequencing

PCR products were generated in a 25 μL reaction volume with 400 nmol/L of forward, 40 nmol/L reverse and 400 nmol/L universal biotinylated primers, 200 μmol/L of each dNTP, 1.5 mmol/L or 2.5 mmol/L MgCl_2 _(see Additional FIle 9 for all primer sequences and reaction conditions), 1x Platinum-Taq reaction buffer and 1.25 units PlatinumTaq™ (Invitrogen, Karlsruhe, Germany). PCR conditions were 95°C for 5 minutes, followed by 45 cycles with denaturation at 95°C for 30 seconds, annealing at 55°C or 60°C for 45 seconds, and elongation at 72°C for 30 seconds finished with 1 cycle final elongation at 72°C for 5 minutes. The reverse primer is tagged by a sequence recognized by the universal primer. Therefore, a single (expansive) biotinylated primer can be used for all different gene-specific assays [21].

PCR products (5-20 μL) were added to a mix consisting of 3 μL Streptavidin Sepharose HP™ (Amersham Biosciences, Freiburg, Germany) and 37 μL binding buffer (Qiagen, Hilden, Germany) and mixed at 1200 rpm for 5 minutes at room temperature.

Using the Vacuum Prep Tool™ (Qiagen, Hilden, Germany), single-stranded PCR products were prepared following the manufacturer's instructions. The sepharose beads with the single stranded templates attached were released into a PSQ 96 Plate Low™ (Qiagen, Hilden, Germany) containing a mix of 12 μL annealing buffer (Qiagen, Hilden, Germany) and 500 nmmol/L of the corresponding sequencing primer (see Additional File 9). Pyrosequencing™ reactions were performed in a PyroMark MD System (Qiagen, Hilden, Germany) according to the manufacturer's instructions using the PyroGold SQA™ Reagent Kit (Qiagen, Hilden, Germany). CpG site quantification was performed using the methylation Software Pyro Q-CpG™.

### Sequence analysis

59,366 reads were obtained from a single run. All the 454 sequences came in one large FASTA file with one sequence read per entry. The sequences were from the forward strand. The primers were composed of a 9-10 nucleotide initial sequence used as a tag to identify the sample. Primary analysis of the sequencing results was conducted using the freely available next-generation sequence software Galaxy (http://main.g2.bx.psu.edu/). Each amplicon sequence was assigned to 1 of the 12 loci under study based on the tagged locus-specific primers. To determine sequence identity, sodium bisulfite conversion efficiency and the methylation state for each CpG site, the amplicon sequences were analyzed by using a web-based freely available quantification tool for methylation analysis (QUMA, [32]). The percent identity scores were set at 90% and CpH conversion efficiency were set at 100%. The sequences were then filtered at 90% sequence identity and 100% CpH conversion efficiency.

Statistical analyses were carried out using Microsoft Excel, GraphPad Prism5 and QUMA [32] software.

For the comparison of the two methods Bland-Altman-Plots were generated [35]. In these plots the difference of two methods is plotted against the average of both methods. A comprehensive description of this type of data presentation by Altman and Bland ("Measurements in Medicine: the Analysis of Method Comparison Studies", The Statistician 32 (1983) 307 - 317) can be found freely available at: https://person.hst.aau.dk/slc/Teaching/Papers/BlandAltman83.pdf

### List of abbreviations

CpH: cytosine followed by adenine, thymine, or cytosine; miR: microRNA; qMSP: quantitative Methylation-specific PCR

## Authors' contributions

UL, AP and HK conceived the study; AP, CA, and BH performed all experiments prior to 454 sequencing; SL established and performed the 454 sequencing under the guidance of SS; AP, CA, and UL analyzed the 454 and the pyrosequencing data; KH performed the statistical analysis; HK selected and evaluated all cases; UL and AP wrote the manuscript with support from HK, BH, and SS. All authors read and approved the final manuscript.

## Supplementary Material

Additional File 1 Number of reads for all loci in all 10 HCC specimens Click here for file

Additional File 2 **Relationship between number of reads per sample and amplicon length, secondary structure formation, and GC content**. Click here for file

Additional File 3 **Bland-Altman Plots for all 12 loci separately**. Click here for file

Additional File 4 **Regression analysis for all 12 loci separately**. Click here for file

Additional File 5 **Detailed description of differences between 454 sequencing and conventional pyrosequencing**. Click here for file

Additional File 6 **Example for details obtained only by 454 sequencing: Exact determination of the number of fully methylated alleles**. Click here for file

Additional File 7 **Example for details obtained only by 454 sequencing: "Heterogeneous" methylation patterns versus dichotomous patterns ("fully methylated or fully unmethylated")**. Click here for file

Additional File 8 **HCC specimens under study**. Click here for file

Additional File 9 **Primer sequences**. Click here for file
