# Supplementary figures and images for: Systematic cross-validation of 454 sequencing and pyrosequencing for the exact quantification of DNA methylation patterns with single CpG resolution
Source: BMC Biotechnol. 2011 Jan 14;11:6. doi: 10.1186/1472-6750-11-6 (PMC3032674; doi:10.1186/1472-6750-11-6)

## Slide 1
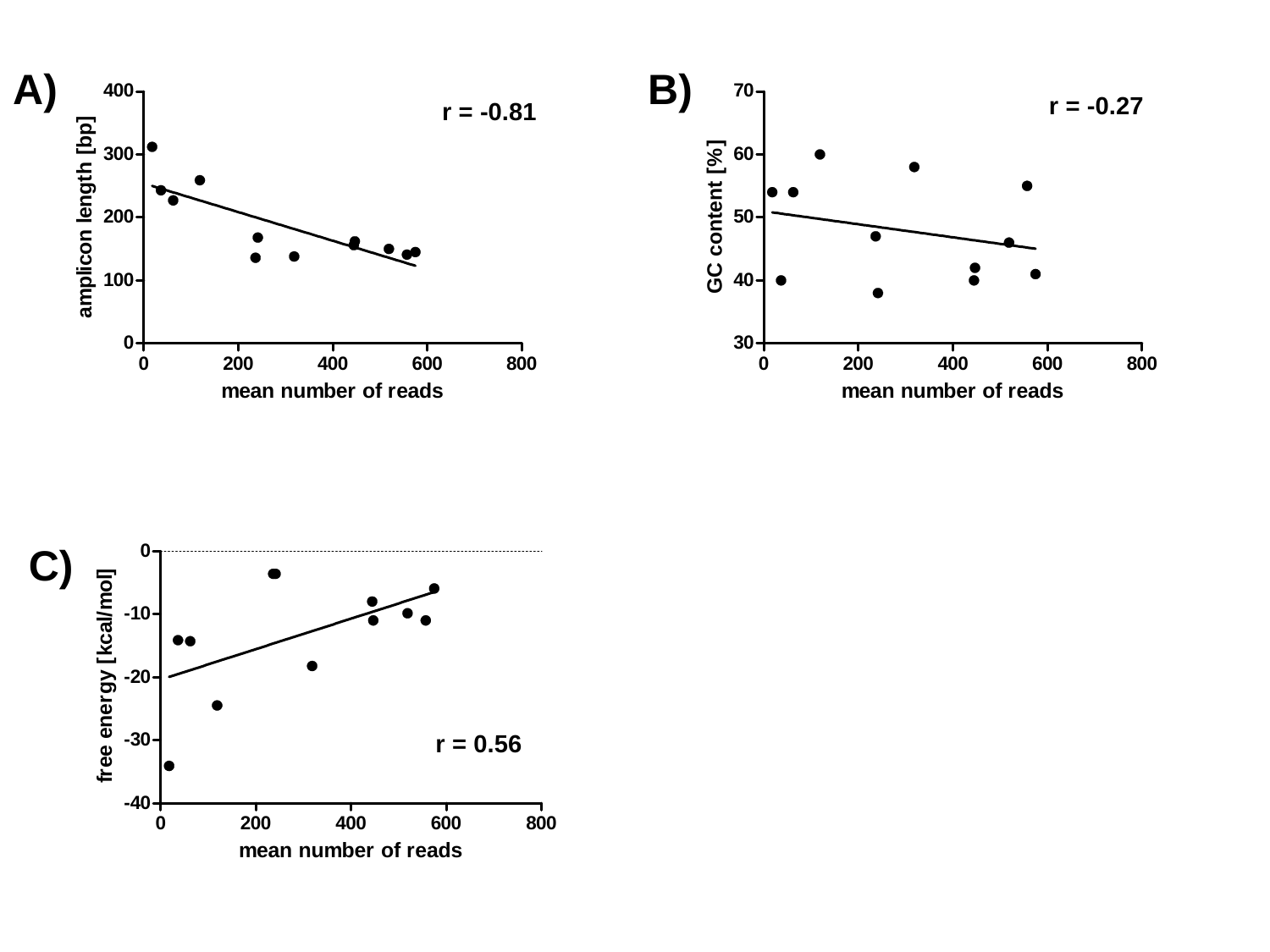

A)
B)
r = -0.27
r = -0.81
C)
r = 0.56

Supplement: Additional File 2 — Relationship between number of reads per sample and amplicon length, secondary structure formation, and GC content. [file 1472-6750-11-6-S2.PPT]

## Slide 1
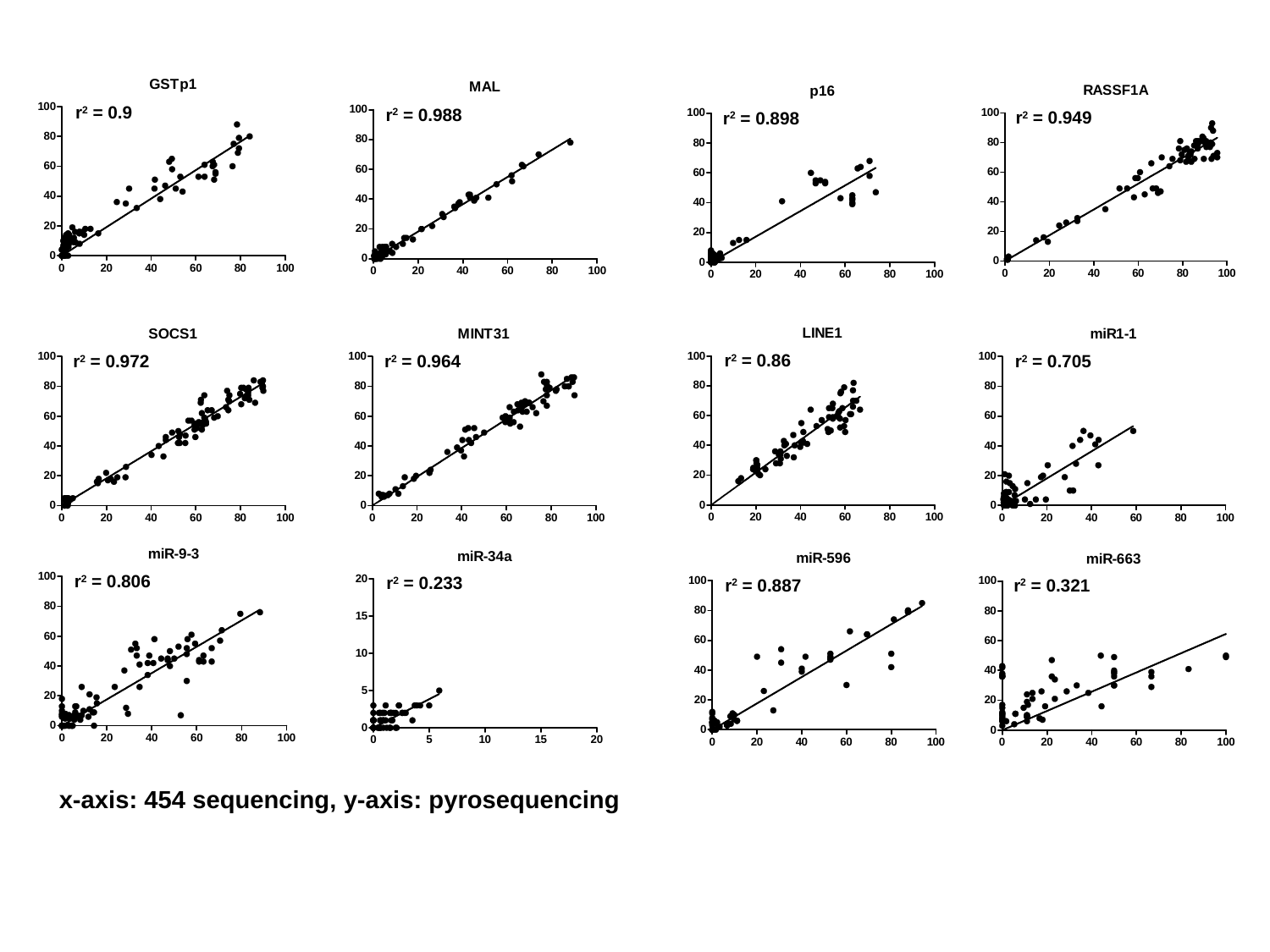

r2 = 0.9
r2 = 0.988
r2 = 0.949
r2 = 0.898
r2 = 0.86
r2 = 0.972
r2 = 0.964
r2 = 0.705
r2 = 0.806
r2 = 0.233
r2 = 0.887
r2 = 0.321
x-axis: 454 sequencing, y-axis: pyrosequencing

Supplement: Additional File 4 — Regression analysis for all 12 loci separately. [file 1472-6750-11-6-S4.PPT]

## Slide 1
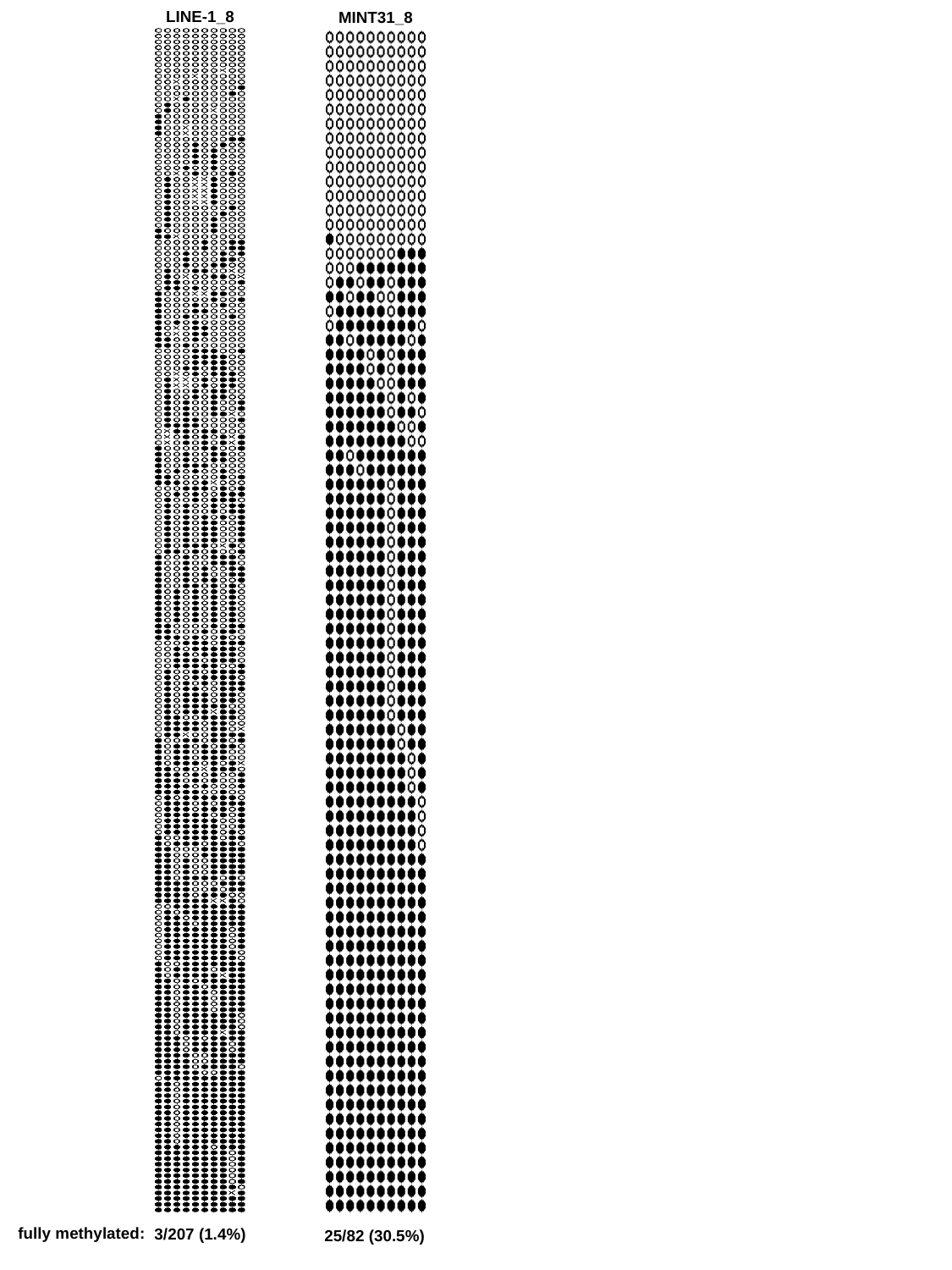

LINE-1_8
MINT31_8
fully methylated:
3/207 (1.4%)
25/82 (30.5%)

Supplement: Additional File 6 — Example for details obtained only by 454 sequencing: Exact determination of the number of fully methylated alleles. [file 1472-6750-11-6-S6.PPT]

## Slide 1
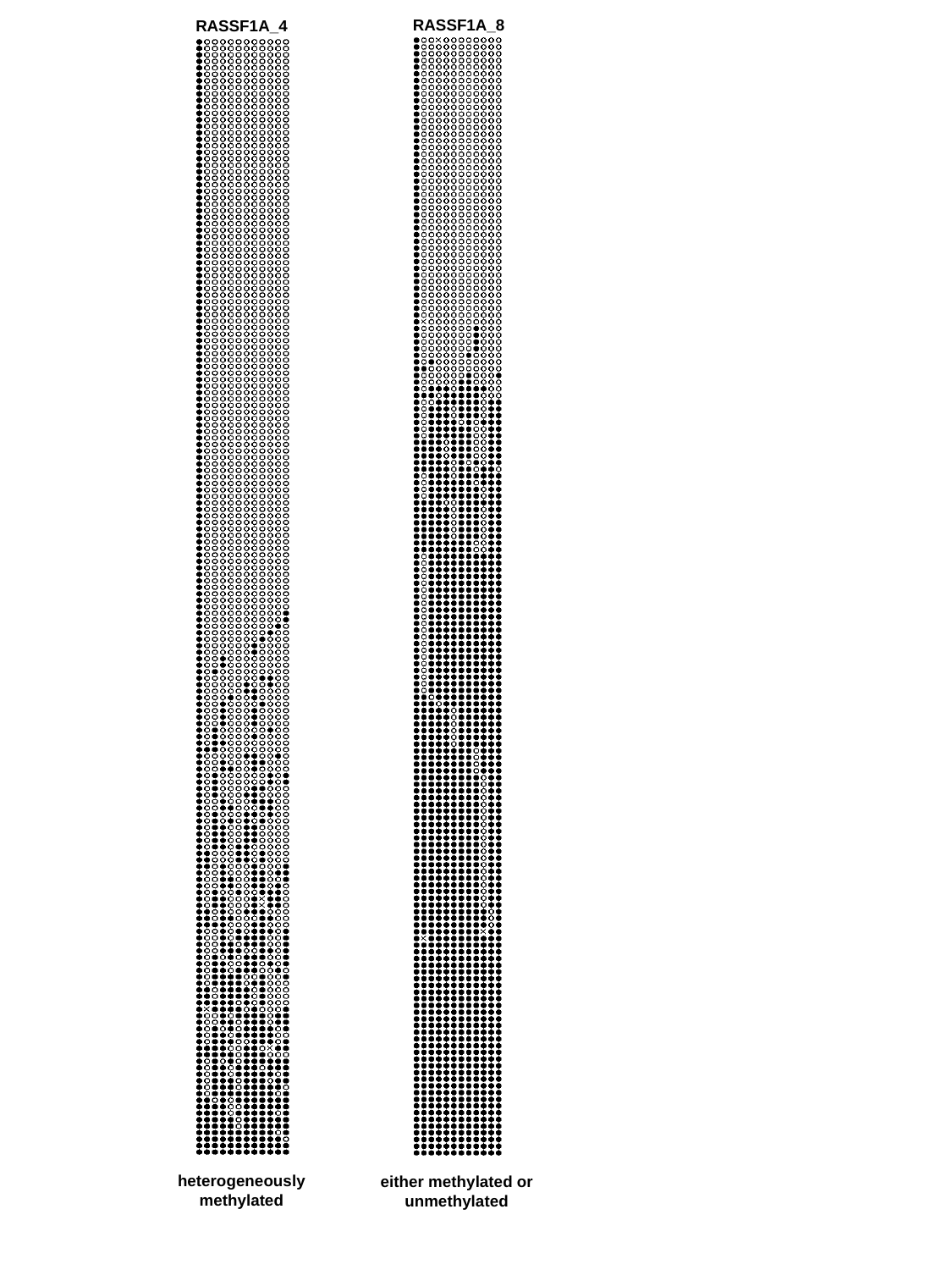

RASSF1A_8
RASSF1A_4
heterogeneously
methylated
either methylated or
unmethylated

Supplement: Additional File 7 — Example for details obtained only by 454 sequencing: "Heterogeneous" methylation patterns versus dichotomous patterns ("fully methylated or fully unmethylated"). [file 1472-6750-11-6-S7.PPT]
