# Supplementary material for: Systematic cross-validation of 454 sequencing and pyrosequencing for the exact quantification of DNA methylation patterns with single CpG resolution
Source: BMC Biotechnol. 2011 Jan 14;11:6. doi: 10.1186/1472-6750-11-6 (PMC3032674; doi:10.1186/1472-6750-11-6)
Supplement: Additional File 3 — Bland-Altman Plots for all 12 loci separately. [file 1472-6750-11-6-S3.PPT]

## Slide 1
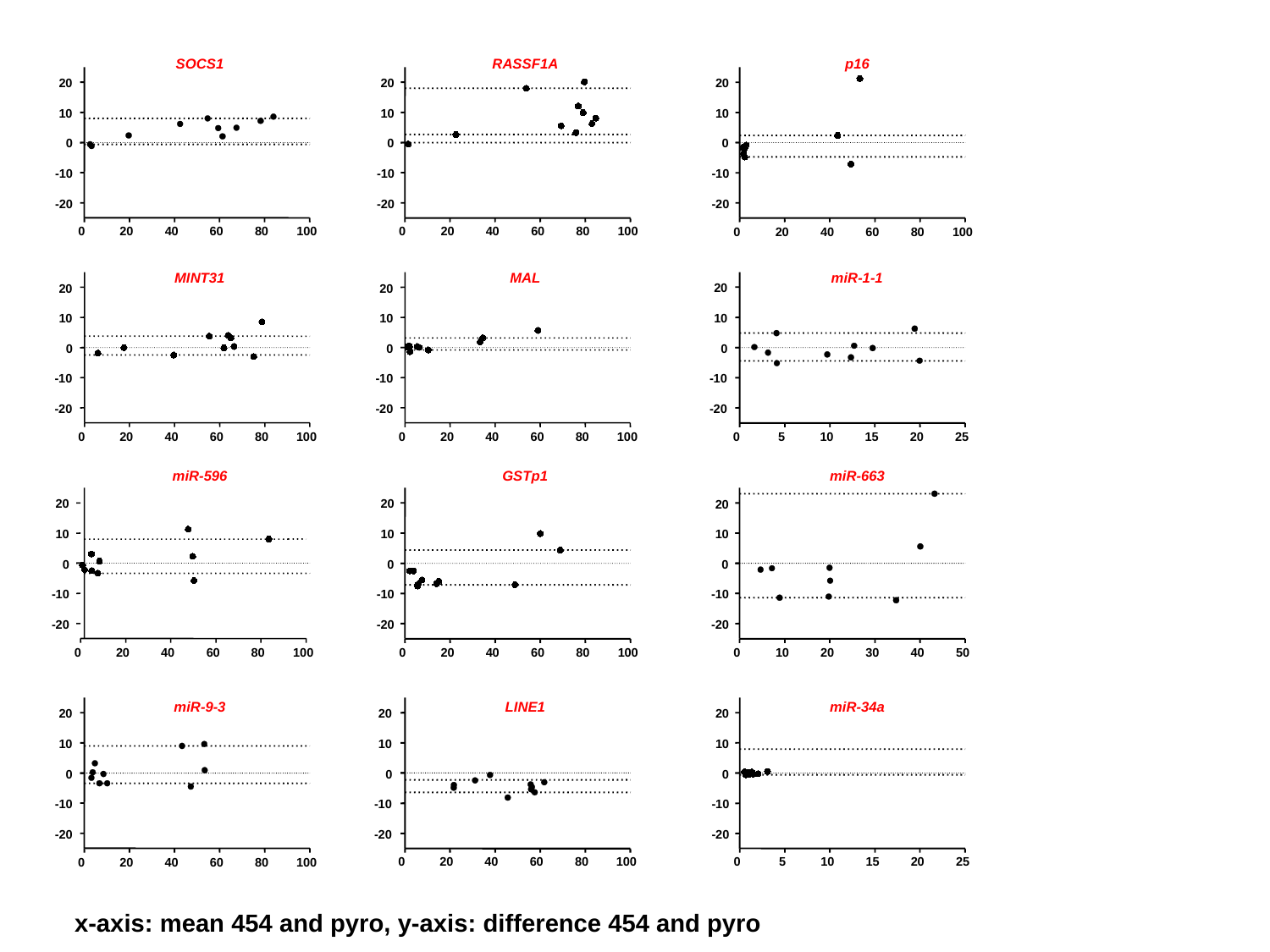

20
10
0
-10
-20
0
20
40
60
80
100
20
10
0
-10
-20
0
20
40
60
80
100
20
10
0
-10
-20
0
20
40
60
80
100
SOCS1
RASSF1A
p16
20
10
0
-10
-20
0
20
40
60
80
100
20
10
0
-10
-20
0
20
40
60
80
100
20
10
0
-10
-20
0
5
10
15
20
25
MINT31
MAL
miR-1-1
20
10
0
-10
-20
0
20
40
60
80
100
20
10
0
-10
-20
0
20
40
60
80
100
20
10
0
-10
-20
0
10
20
30
40
50
miR-596
GSTp1
miR-663
20
10
0
-10
-20
0
20
40
60
80
100
20
10
0
-10
-20
0
20
40
60
80
100
20
10
0
-10
-20
0
5
10
15
20
25
miR-9-3
LINE1
miR-34a
x-axis: mean 454 and pyro, y-axis: difference 454 and pyro
