# Supplementary material for: Systematic cross-validation of 454 sequencing and pyrosequencing for the exact quantification of DNA methylation patterns with single CpG resolution
Source: BMC Biotechnol. 2011 Jan 14;11:6. doi: 10.1186/1472-6750-11-6 (PMC3032674; doi:10.1186/1472-6750-11-6)
Supplement: Additional File 5 — Detailed description of differences between 454 sequencing and conventional pyrosequencing. [file 1472-6750-11-6-S5.DOC]

**Additional Table 2**

**Differences in methylation level of individual CpG sites obtained by pyrosequencing and 454 sequencing**

The differences in percentage points were calculated and categorized as indicated. The numbers indicate how many individual measurements fall in each category.

| diff. pyro-454 | **SOCS1** | **RASSF1A** | **p16** | **MINT31** | **MAL** | **miR-1-1** | **miR-596** | **miR-34a** | **GSTp1** | **miR-663** | **miR-9-3** | **LINE-1** | **total** |  |
| --- | --- | --- | --- | --- | --- | --- | --- | --- | --- | --- | --- | --- | --- | --- |
| [percentage points] |  |  |  |  |  |  |  |  |  |  |  |  | 883 |  |
| ≤2 | 22 | 15 | 23 | 26 | 48 | 24 | 16 | 57 | 13 | 3 | 25 | 17 | 289 | 33.1% |
| >2, ≤ 5 | 25 | 9 | 29 | 22 | 24 | 16 | 21 | 3 | 21 | 8 | 26 | 22 | 226 | 26.0% |
| >5, ≤ 10 | 31 | 18 | 9 | 17 | 6 | 19 | 14 | 0 | 38 | 18 | 18 | 18 | 206 | 23,7% |
| >10, ≤ 15 | 11 | 14 | 2 | 4 | 2 | 5 | 4 | 0 | 16 | 12 | 6 | 8 | 86 | 9.7% |
| >15, ≤ 25 | 1 | 13 | 6 | 1 | 0 | 6 | 1 | 0 | 2 | 2 | 13 | 5 | 53 | 5.8% |
| >25 | 0 | 1 | 1 | 0 | 0 | 0 | 4 | 0 | 0 | 6 | 2 | 0 | 23 | 1.6% |
