# Supplementary material for: Systematic cross-validation of 454 sequencing and pyrosequencing for the exact quantification of DNA methylation patterns with single CpG resolution
Source: BMC Biotechnol. 2011 Jan 14;11:6. doi: 10.1186/1472-6750-11-6 (PMC3032674; doi:10.1186/1472-6750-11-6)
Supplement: Additional File 8 — HCC specimens under study. [file 1472-6750-11-6-S8.DOC]

**Additional Table 1**

**Patient Information**

| **Patient** | **Diagnosis** | **Gender** | **Age** |
| --- | --- | --- | --- |
| 1 | HCC | m | 73 |
| 2 | HCC | f | 37 |
| 3 | HCC | m | 70 |
| 4 | HCC | m | 64 |
| 5 | HCC | m | 61 |
| 6 | HCC | m | 63 |
| 7 | HCC | m | 59 |
| 8 | HCC | m | 45 |
| 9 | HCC | f | 34 |
| 10 | HCC | f | 59 |
